# IgG4‐Related Sclerosing Mediastinitis Mimicking Thymoma: A Case Report

**DOI:** 10.1002/rcr2.70379

**Published:** 2025-10-14

**Authors:** Eitetsu Koh, Yasuo Sekine, Tadao Nakazawa, Kenzo Hiroshima

**Affiliations:** ^1^ Department of Thoracic Surgery Tokyo Women's Medical University Yachiyo Medical Center Yachiyo Japan; ^2^ Department of Pathology Tokyo Women's Medical University Yachiyo Medical Center Yachiyo Japan

**Keywords:** case report, IgG4‐related disease, mediastinum, sclerosing inflammation, thymoma

## Abstract

IgG4‐related disease (IgG4‐RD) rarely presents as an anterior mediastinal mass and may radiologically resemble thymic neoplasms. We report a 70‐year‐old man with an incidentally detected anterior mediastinal tumour on chest CT. The lesion measured 33 mm, was well‐circumscribed, homogeneous and partially calcified. Tumour markers and serum IgG4 were within the normal range. The patient underwent thoracoscopic resection under the diagnosis of thymoma. Histopathological examination revealed dense lymphoplasmacytic infiltration and abundant IgG4‐positive plasma cells, confirming IgG4‐related sclerosing mediastinitis. No corticosteroid therapy was administered, and the patient remained disease‐free over 3 years of follow‐up. This case highlights the diagnostic challenge of mediastinal IgG4‐RD and the importance of considering it in the differential diagnosis of thymic tumours, even when serum IgG4 is normal.

## Graphical abstract

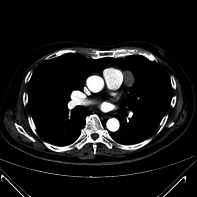

## 1 Introduction

IgG4‐related disease (IgG4‐RD) is a systemic fibroinflammatory condition of unknown aetiology characterised by dense lymphoplasmacytic infiltration, storiform fibrosis and abundant IgG4‐positive plasma cells [1]. While commonly affecting the pancreas, salivary glands and retroperitoneal tissues, involvement of the thoracic region is relatively rare. In particular, isolated lesions in the anterior mediastinum are extremely uncommon and often radiologically mimic thymic neoplasms or lymphomas [2]. Due to the lack of disease‐specific imaging features, preoperative diagnosis of mediastinal IgG4‐RD remains challenging and often requires histopathological evaluation following surgical resection.

Although several cases of thoracic IgG4‐RD have been described in the literature, reports of anterior mediastinal involvement are extremely limited, and many were initially misdiagnosed as thymic epithelial tumours [2, 3]. The overlapping radiological features—such as well‐demarcated margins, homogeneous internal density and occasional calcification—make it difficult to distinguish IgG4‐related sclerosing mediastinitis from more common anterior mediastinal masses. Furthermore, serum IgG4 levels may not always be elevated, and non‐invasive diagnostic criteria are not well established for mediastinal presentations [4]. Consequently, surgical resection is often performed under the presumptive diagnosis of thymoma or other neoplastic conditions, with the definitive diagnosis being made only upon histopathological and immunohistochemical analysis.

For clinically resectable anterior mediastinal masses with a high likelihood of thymoma, major guidelines do not mandate pre‐treatment biopsy because capsule violation may seed tumour cells; a transpleural approach to biopsy should be avoided. When thymoma is confirmed, the oncologic objective is complete excision with total thymectomy. These recommendations are aligned with international guidance stating that pre‐treatment biopsy is not required if upfront resection is feasible and a thymic epithelial tumour is highly probable.

## 2 Case Report

A 70‐year‐old Japanese man was referred to our hospital following the incidental detection of an anterior mediastinal mass on routine chest radiography during a health checkup. He had no symptoms such as chest pain, cough or dyspnoea. His medical history included type 2 diabetes mellitus. He was an ex‐smoker with a 42 pack‐year history (20 cigarettes/day from age 20 to 62). He had no known history of autoimmune disease or allergies, and his family history was unremarkable.

On physical examination, the patient was afebrile with stable vital signs. No lymphadenopathy or abnormal findings were noted on auscultation. Laboratory tests, including liver and renal function, complete blood count and coagulation profile, were all within normal limits. Tumour markers, including carcinoembryonic antigen (CEA), cytokeratin 19 fragment (CYFRA 21‐1) and alpha‐fetoprotein (AFP), were also normal. Pulmonary function tests and electrocardiography revealed no abnormalities.

**FIGURE 1:**
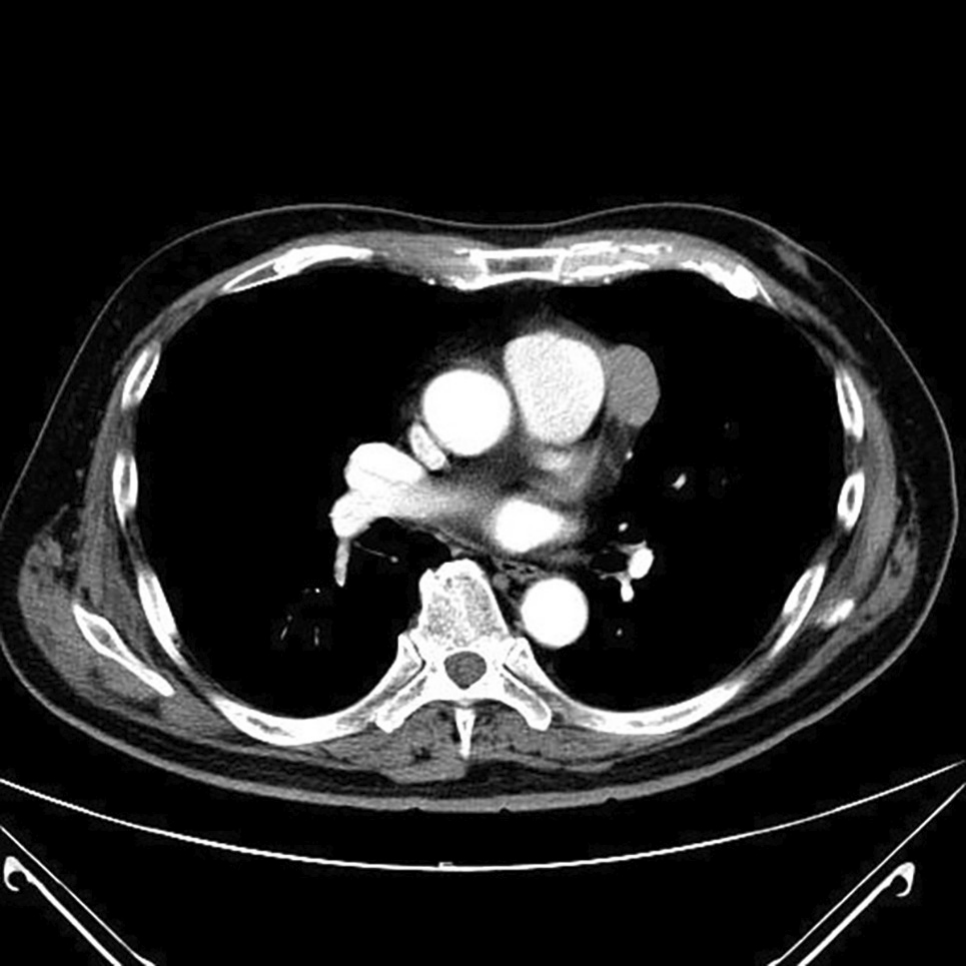
Axial contrast‐enhanced computed tomography (CT) image of the chest showing a 33‐mm well‐defined, homogeneous mass with partial calcification located in the left anterior mediastinum adjacent to the main pulmonary artery. The lesion was initially suspected to be a thymoma.

Contrast‐enhanced computed tomography (CT) of the chest revealed a 33‐mm, well‐defined, homogeneous mass with partial calcification located in the left anterior mediastinum adjacent to the main pulmonary artery (Figure 1). There was no evidence of invasion into surrounding structures or lymphadenopathy. Based on these imaging findings, a preoperative diagnosis of thymoma was made. In keeping with contemporary guidance that pre‐treatment biopsy is not required when a resectable thymic epithelial tumour is strongly suspected—and that a transpleural approach should be avoided if biopsy is pursued—we did not obtain a preoperative biopsy [1, 2].

Operative strategy at our centre is to plan total thymectomy for a lesion highly suspicious for thymoma, with intraoperative readiness to convert to limited thymic resection only if the mass is not in macroscopic continuity with the thymus and an en bloc R0 resection can be achieved without capsule violation. In this patient, the mass was embedded in anterior mediastinal fat without gross thymic continuity or invasion of adjacent structures; an en bloc thoracoscopic limited thymic resection with a cuff of thymic tissue and mediastinal fat was performed, achieving negative margins.

**FIGURE 2:**
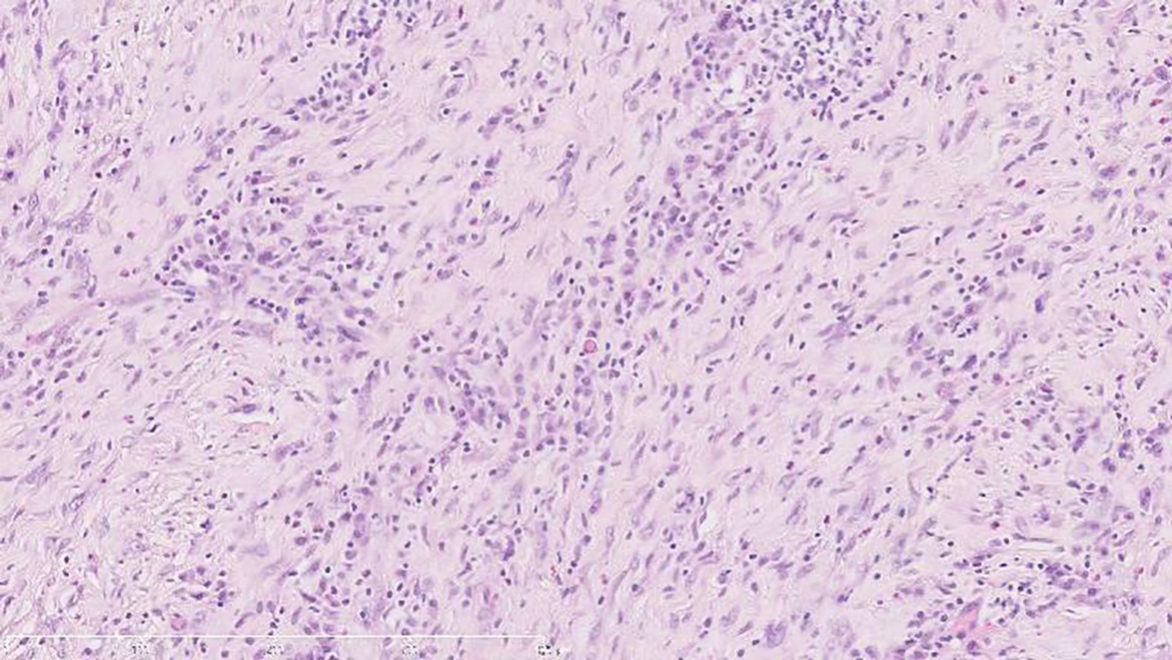
Haematoxylin and eosin (H&E) staining of the mediastinal mass. Dense lymphoplasmacytic infiltration within fibrotic stroma is evident, with numerous plasma cells. (Original magnification ×200).

**FIGURE 3:**
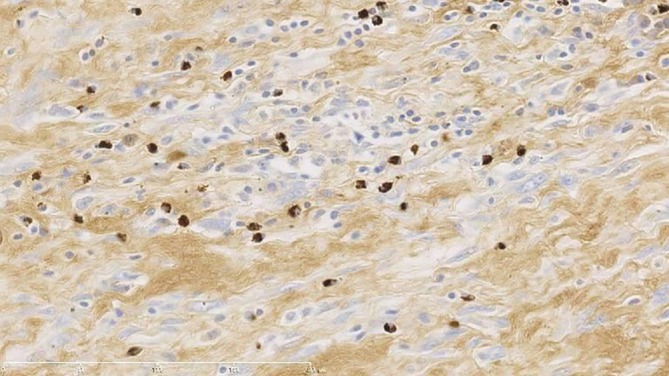
Immunohistochemical staining for IgG4. A significant number of IgG4‐positive plasma cells are seen, consistent with the diagnosis of IgG4‐related sclerosing mediastinitis. In undiagnosed anterior mediastinal masses in which malignancy remains possible, percutaneous biopsy carries specific risks: (i) pleural tumour seeding—particularly relevant if the lesion represents a thymic epithelial tumour—and (ii) rare but serious air embolism due to proximity to the great vessels and cardiac structures. Accordingly, in resectable cases with high clinical suspicion for thymic epithelial tumour, upfront surgical resection is widely regarded as a standard diagnostic and therapeutic strategy, consistent with major guidelines [5, 6]. Moreover, mediastinal involvement of IgG4‐related disease is rare, and preoperative recognition is challenging; therefore, definitive diagnosis frequently depends on surgical histopathology and immunohistochemistry.

Histopathological examination revealed dense lymphoplasmacytic infiltration within fibrotic stroma. Numerous plasma cells were observed, many of which displayed features suggestive of reactivity, including binucleation and large cell size. Immunohistochemical staining demonstrated that the majority of IgG‐positive plasma cells were also positive for IgG4, consistent with a diagnosis of IgG4‐related sclerosing mediastinitis. Post‐operative serum IgG4 level was 89.3 mg/dL (normal range: 4.8–105 mg/dL), while IgG1 was slightly elevated at 806 mg/dL (normal range: 320–748 mg/dL). No corticosteroid therapy was initiated. The patient has been followed for 3 years without evidence of recurrence or new organ involvement (Figures 2 and 3).

## 3 Discussion

This case highlights two important clinical observations. First, IgG4‐RD can manifest as an anterior mediastinal mass that closely mimics thymoma both radiologically and clinically, making preoperative diagnosis particularly difficult. Second, the diagnosis of IgG4‐RD may still be established histopathologically even when serum IgG4 levels are within the normal range. These findings underscore the need for clinicians to consider IgG4‐RD in the differential diagnosis of mediastinal tumours, particularly in cases with atypical features or uncharacteristic clinical presentation.

Regarding preoperative tissue diagnosis, major guidelines state that surgical biopsy should be avoided when a resectable thymoma is strongly suspected, and if a biopsy is pursued, a transpleural approach should be avoided to minimise the risk of pleural seeding [5]. Consistently, the SEOM/GECP/GETTHI guideline notes that baseline biopsy is not required if there is a high suspicion of thymic epithelial tumour and upfront resection is achievable [6]. Our decision not to perform a preoperative biopsy was aligned with these recommendations.

Practice patterns vary by institution. For proven thymoma, most guidelines recommend total thymectomy; however, when a small, well‐encapsulated lesion is not in macroscopic continuity with the thymus and complete en bloc removal is feasible, some centres (including ours) may perform a limited thymic resection. We acknowledge this as a limitation of our case and have clearly described our intraoperative decision‐making to avoid capsule violation and achieve R0 resection.

As to earlier diagnosis without surgery, when a safe and on‐path route exists, an image‐guided core‐needle biopsy via an extrapleural anterior/parasternal approach or a small anterior mediastinotomy can be considered, with tissue processing for IgG4 and total IgG immunohistochemistry and assessment for storiform fibrosis and obliterative phlebitis. Systemic evaluation for other organ involvement of IgG4‐RD may increase diagnostic confidence; however, serologic markers (including serum IgG4) are insufficiently reliable. FDG‐PET uptake is variable and non‐specific. In small, resectable anterior mediastinal masses with high suspicion for thymic epithelial tumour and uncertain biopsy yield or safety, diagnostic surgery remains a reasonable approach to obtain definitive histology and therapy in one procedure.

This case adds to the limited but growing body of literature on IgG4‐RD involving the mediastinum. Awareness of this entity may prevent unnecessary extensive surgery or overtreatment, especially if preoperative biopsy is feasible and likely to be diagnostic; however, definitive confirmation typically depends on permanent‐section immunohistochemistry. Future studies may help to clarify the prevalence, optimal diagnostic pathways and long‐term outcomes of thoracic IgG4‐RD.

## Consent

Written informed consent for publication was obtained from the patient using the official Respirology Case Reports patient consent form.

## Conflicts of Interest

The authors declare no conflicts of interest.

## Data Availability

The data that support the findings of this study are available on request from the corresponding author. The data are not publicly available due to privacy or ethical restrictions.
